# Development of a novel rodent model for dog heartworm microfilaremia using the severe-combined immunodeficiency mouse

**DOI:** 10.1038/s41598-024-63165-x

**Published:** 2024-06-14

**Authors:** Mihoko Mizuseki, Nao Ikeda, Takahiro Shirozu, Maki Yamagishi, Sugao Oshiro, Shinya Fukumoto

**Affiliations:** 1 National Research Center for Protozoan Diseases, Obihiro University of Agriculture and Veterinary Medicine, Inada-cho, Obihiro, Hokkaido 080-8555 Japan; 2 Yanbaru Animal Clinic, Nago, Okinawa 905-0019 Japan

**Keywords:** *Dirofilaria immitis*, Microfilaria, Mouse model, Parasitology, Microbiology techniques

## Abstract

*Dirofilaria immitis* is a mosquito-borne parasitic nematode that causes fatal heartworm disease in canids. The microfilariae are essential for research, including drug screening and mosquito-parasite interactions. However, no reliable methods for maintaining microfilaria long-term are currently available. Therefore, we used severe combined immunodeficiency (SCID) mice to develop a reliable method for maintaining *D. immitis* microfilaria. SCID mice were injected intravenously with microfilariae isolated from a *D. immitis*-infected dog. Microfilariae were detected in blood collected from the tail vein 218 days post-inoculation (dpi) and via cardiac puncture 296 dpi. Microfilariae maintained in and extracted from SCID mice showed infectivity and matured into third-stage larvae (L3s) in the vector mosquito *Aedes aegypti.* L3s can develop into the fourth stage larvae in vitro. Microfilariae from SCID mice respond normally to ivermectin in vitro. The microfilariae in SCID mice displayed periodicity in the peripheral circulation. The SCID mouse model aided in the separation of microfilariae from cryopreserved specimens. The use of SCID mice enabled the isolation and sustained cultivation of microfilariae from clinical samples. These findings highlight the usefulness of the SCID mouse model for studying *D. immitis* microfilaremia in canine heartworm research.

## Introduction

*Dirofilaria immitis* is one of the most prevalent parasitic nematodes in veterinary medicine. In dogs and other members of the Canidae, this parasite causes a fatal illness known as heartworm disease^1,2^. Dogs with a *D. immitis* infection show severe clinical symptoms. Coughing and exercise intolerance are the first signs; however, subsequent hemodynamic disturbances result in pulmonary hypertension and cardiac hypertrophy^1^. When there is an obstruction in the blood flow via the lungs, numerous adult worms in the pulmonary arteries may result in heart failure^1^. Notably, *D. immitis* tends to infect not only dogs, but also cats, humans, and a variety of mammalian taxa^2^. This zoonotic illness presents as dirofilariasis in human hosts, which is characterized by pulmonary nodule formation^3^.

The lifecycle of *D. immitis* includes an intermediate host, the mosquito, and a definitive host, the canid^1^. Adult female worms release microfilariae into their bloodstream. Microfilariae are picked up by the vector mosquito while it is taking a blood meal, thus infecting it. Following the mosquito’s subsequent blood meal, third-stage larvae (L3) in the proboscis infiltrate a new mammalian host, developing into fourth-stage larvae (L4) and immature adults before maturing into adult worms. These adult worms parasitize the pulmonary arteries and continue to produce microfilariae for years.

The primary method of preventing *D. immitis* infection is the administration of macrocyclic lactone anthelmintics, such as ivermectin, milbemycin oxime, and moxidectin^4^. These anthelmintics are usually administered at 30-day intervals throughout the active mosquito season to prevent heartworm disease^1^. However, the advent of macrocyclic lactone resistance in *D. immitis* has raised substantial concern^5^. Phenotypic resistance to macrocyclic lactones in *D. immitis* has primarily been observed in North America^1,5,6^. The global rise of macrocyclic lactone resistance is concerning, highlighting the need for novel anthelmintic classes and strategies to control heartworm disease.

The availability of microfilaria is crucial for both assessing macrocyclic lactone resistance in *D. immitis* and screening new drugs^7^. Initial assays are commonly conducted using microfilaria because of their convenience^6,8^. Microfilariae are also necessary to obtain L3s from mosquitoes for macrocyclic lactone resistance assays because the L3 and early L4 are prime targets for anthelmintic prophylaxis^5^.

Furthermore, blocking transmission at the mosquito stage is regarded as a novel and useful strategy for controlling heartworm disease. Consequently, a detailed understanding of the *D. immitis* transmission mechanisms in mosquito vectors is essential for such research projects. In this context, a steady supply of microfilariae is critical because damaged microfilariae, such as cryopreserved microfilariae, cannot infect mosquitoes normally^9^.

The original method for preserving and isolating microfilariae required the use of infected dogs, which was expensive, time-consuming, and necessitated a well-equipped laboratory. Moreover, the use of numerous dogs raises ethical concerns about animal welfare particularly when isolating and maintaining multiple heartworm strains from clinical samples. Alternatively, microfilariae can be preserved through in vitro culture and cryopreservation. However, in vitro culture can only sustain *D. immitis* microfilariae for less than 2 months^10^, and cryopreservation substantially reduces the viability of recovered microfilariae compared to fresh microfilaria^9,11^. The rodent microfilaria model is regarded as a viable alternative to *D. immitis*-infected dogs. Alternatively, a gamma-irradiated mouse model has been used for *D. immitis* microfilaremia^12^, albeit for a short period of only five weeks. As a result, there is an urgent need for a new technique to maintain microfilariae.

In the present study, we developed a sustainable mouse model to maintain *D. immitis* microfilariae without the need for dogs. We validated the microfilariae transplantation technique on severely combined immunodeficient (SCID) mice. We determined how long microfilariae could be maintained in SCID mice and if they preserved their mosquito infectivity. Furthermore, we investigated whether microfilariae grown in SCID mice could be used for drug tests by isolating and recovering cryopreserved microfilariae.

## Materials and methods

### Animals

We used SPF C.B-17/Icr-scid/scid (SCID) and BALB/cA (BALB/c) mice in this study (CLEA, Tokyo, Japan). Mice were housed under standard conditions at 22 °C with a light cycle of 08:00 to 20:00 h. A 6-month-old female beagle dog (KITAYAMA LABES Co., Nagano, Japan) was subcutaneously infected with 40 L3s of the *D. immitis* SF1 strain, initially isolated in Japan^9,13^ from the dorsal neck region*.* The dog was housed in an individual cage under standard conditions at 23 °C with a light cycle of 06:00 to 18:00 h. The *Aedes aegypti* Liverpool-OB strain, susceptible to *D. immitis* infection^9^, was kept at 27 °C and > 80% humidity on a diet of 5% fructose solution supplemented with 0.05% para-aminobenzoic acid (PABA) under a 12-h light and 12-h dark photoperiod. All animal experiments complied with the Guide for Laboratory Animals of the Obihiro University of Agriculture and Veterinary Medicine and in accordance with ARRIVE guidelines. The Committee of Animal Experiments of the Obihiro University of Agriculture and Veterinary Medicine (permit number 22-123) approved the protocol.

### Purification and cryopreservation of microfilariae

A heparinized syringe was used to collect blood from the cephalic vein of a dog infected with *D. immitis* and which was microfilaremic. Microfilariae were purified using a filtration method^14^. Blood was diluted 1:10 with RPMI Medium 1640 (Thermo Fisher Scientific, Waltham, MA, USA) containing penicillin and streptomycin (Thermo Fisher Scientific). A Swinnex Filter Holder 25 mm (Merck, Darmstadt, Germany) was assembled with an Isopore 5.0 µm PC Membrane (Merck). The diluted blood was filtered, and the filter unit was washed with fresh RPMI medium. The filter membrane was placed in a 35-mm Petri dish with 400 µL of medium to release the captured microfilariae. To collect the discharged microfilariae, we centrifuged them at 1000×*g* for 10 min at 4 °C. We also used a clinical isolate of microfilariae derived from naturally infected canine blood which had been collected at a veterinary clinic in Nago, Okinawa, Japan. The *D. immitis* clinical isolate was named ON1. Microfilariae isolated from the *D. immitis*-infected dog were cryopreserved as described by Shirozu, Soga, and Fukumoto^9^. This study used cryopreserved microfilariae that had been stored for more than 3 months.

### Inoculation of microfilariae into mice and determination of microfilaremia

Microfilariae isolated from experimentally infected dogs or cryopreserved were used within hours of blood collection. Clinical ON1 microfilariae were injected into one SCID mouse three days after blood collection. The blood was transported and stored at 4 °C until needed. Microfilariae were resuspended in 200 µL of RPMI medium 1640 and inoculated into the mouse tail vein. Some mice underwent splenectomy under isoflurane anesthesia and were given at least one week to adapt. In high microfilariae dose test, two SCID mice were inoculated with 1.9 × 10^5^ of *D. immitis* microfilariae via tail vein. To evaluate peripheral blood microfilaremia, 10 µL of blood was collected from the tail vein every week between 18:00 and 20:00 and resuspended in RPMI medium 1640. The microfilariae were counted using an inverted microscope (Eclipse TS100, Nikon, Tokyo, Japan). When microfilariae were no longer found in the peripheral blood, cardiac blood taken via cardiac puncture under anesthesia was used to measure the number of microfilariae, and each experiment was terminated. Microfilaremia levels were measured every three hours, from 8:00 to 20:00 to establish periodicity. Microfilaremia in a *D. immitis*-infected dog, which served as the source of microfilariae inoculated into mice, was also monitored every three hours between 8:00 and 20:00. Microfilariae were kept in SCID mice for at least two months before being used in subsequent experiments throughout this study.

### Microfilaria infectivity assay to the mosquito

On days 5–8 post-emergence, female *Ae. aegypti* mosquitoes were fed blood containing microfilariae for 1 h using an artificial membrane-feeding system with Parafilm as the membrane (Bemis Company Inc., Neenah, WI, USA). The concentration of microfilariae was adjusted to one to four microfilariae per microliter using heparinized uninfected dog blood. Following blood feeding, fully-fed mosquitoes were separated under CO_2_ anesthesia and used for further study. To count the number of L3 larvae, all mosquitoes were dissected under a stereomicroscope 13 days post-infection^15^.

### Fourth-stage larva (L4) development assay

L3 larvae isolated from *D. immitis*-infected mosquito heads were used at day 13 post-infection (dpi)^15^. Ten L3 parasites were cultured in 24-well culture plates containing 1 mL of RPMI Medium 1640 with 20% fetal bovine serum (FBS) (BioWest, Nuaillé, France), 10% glucose (Wako Pure Chemical Industries, Ltd., Osaka, Japan), and penicillin–streptomycin for nine days at 37 °C in a 5% CO_2_ atmosphere. The number of L4 larvae was counted daily using the molting shell number^16^. Each experiment was carried out in triplicate.

### Microfilariae in vitro cultivation and drug sensitivity assay

Five hundred microfilariae per well were cultured in 24-well flat-bottom plates using RPMI Medium 1640 with 10% FBS and penicillin–streptomycin at 37 °C in an atmosphere of 5% CO_2_. The survival of the microfilariae was determined by counting 200 microfilariae per well weekly using an inverted microscope. Each experiment was performed in triplicate. The drug sensitivity assay used ivermectin (FUJITA PHARM, Tokyo, Japan) at concentrations ranging from 3.125 to 50.0 mM. Five hundred microfilariae per well were cultured with the addition of each concentration of ivermectin in 24-well flat-bottom plates using RPMI Medium 1640 with 10% FBS and penicillin–streptomycin. Each experiment was performed in triplicate. Microfilarial survival was assessed daily, as described above.

### Statistical analysis

All statistical analyses were conducted using GraphPad Prism software version 10 (GraphPad, La Jolla, CA, USA). Two group comparison of microfilaria survival, blood microfilariaemia, number of the L3 in individual mosquito and L3 to L4 molting assay were analyzed by Mann–Whitney test. Microfilariae survival in ivermectin response assay was analyzed by unpaired t-test. Microfilariae periodicity and three groups comparison of L3 number were analyzed by Kruskal–Wallis test. Three groups comparison of L3 to L4 molting assay was analyzed by one-way ANOVA. A *p* value less than 0.05 was considered statistically significant.

## Results

### The SCID mouse is an invaluable tool for the rodent model of *D. immitis*

**Figure 1:**
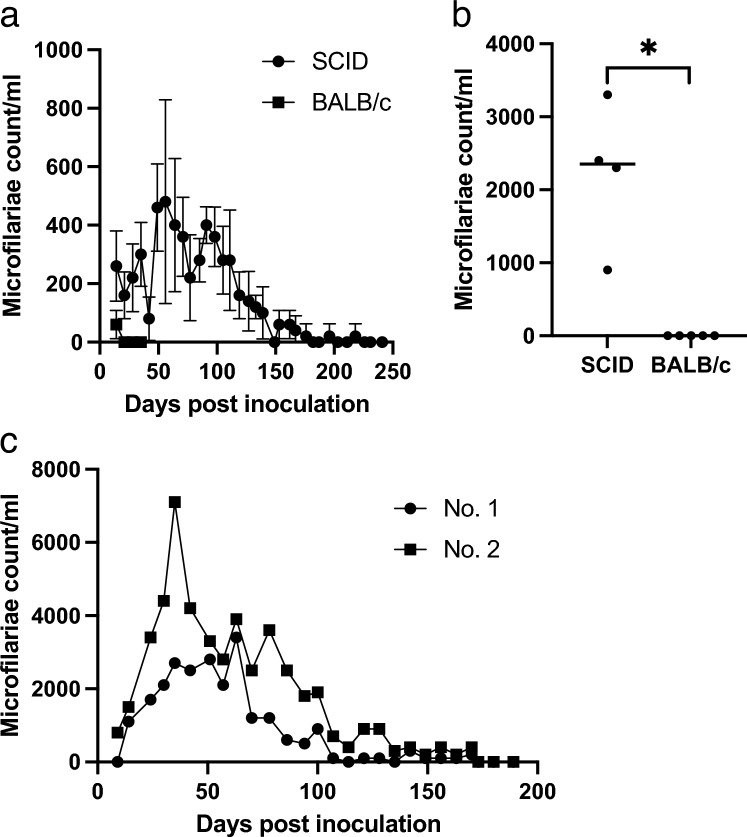
Long-term *D. immitis* microfilariaemia in a rodent model using the SCID mouse. (**a**) Comparison of the peripheral blood microfilaremia period between SCID mice and BALB/c mice. Mice (n = 5) were inoculated with 3.0 × 10^4^ microfilariae via the tail vein, and peripheral blood microfilaremia was monitored every week. SCID mice showed significantly prolonged microfilaremia (Mann–Whitney test, *p* < 0.01). Error bars = mean ± SD. (**b**) Microfilaremia count of the blood collected by cardiac puncture of the individual mice. Cardiac puncture was done after peripheral blood microfilariae had disappeared. *p < 0.01 (Mann–Whitney test). (**c**) High microfilariae dose test. Microfilaremia of the SCID mice inoculated with a high dose (1.9 × 10^5^) of *D. immitis* microfilariae (n = 2). *SCID* severe combined immunodeficiency.

**Figure 2:**
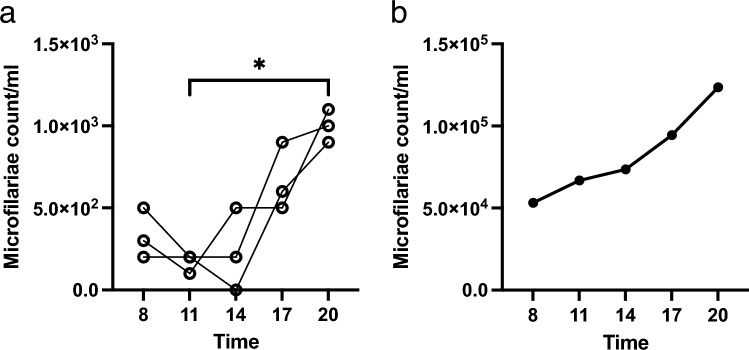
Periodicity of microfilaremia in the SCID mice. The time course of peripheral microfilaremia in individual SCID mice (n = 3) (**a**) and a dog (**b**) in a day was monitored every 3 h between 8 a.m. and 8 p.m. SCID mice were used at least 3 months after the inoculation of microfilariae. *p < 0.05 (Kruskal–Wallis test). *SCID* severe combined immunodeficiency.

SCID and BALB/c mice were inoculated with purified microfilariae. The microfilaremia in SCID mice was significantly longer than that in the control BALB/c mice (*p* = 0.0079). Microfilaremia was detected in the peripheral blood of SCID mice until 218 days post-infection (dpi) (median, 176 dpi) (Fig. 1a) and 296 dpi in the cardiac blood (Fig. 1b). In contrast, microfilaremia was only detected in the peripheral blood of control BALB/c mice until 14 dpi (median, 14 dpi). When the mice were euthanized 35 days after the peripheral microfilaria disappeared, there were no microfilaria in the cardiac blood (Fig. 1b). During the early phase of infection in the high microfilaria dose inoculation test, there were many microfilariae in the peripheral blood. However, the microfilaremia period was short and disappeared by 175 dpi (Fig. 1c). We also investigated the effect of splenectomy on microfilaremia in mice using two sets of experiments. However, no significant difference was found in SCID mice, and there was no reproducible difference in the microfilaremia duration between the splenectomized and non-splenectomized groups (data not shown). To determine whether microfilaremia in SCID mice exhibited periodicity, we monitored the changes in microfilaremia throughout the circadian cycle. SCID mice showed significantly higher microfilaremia at 20:00 (*p* = 0.0477, Fig. 2a). The peak time of microfilaremia in SCID mice was comparable to that in source dog (Fig. 2b).

### The microfilariae maintained in SCID mice retained the ability to mature to the L3 and L4 stages

**Figure 3:**
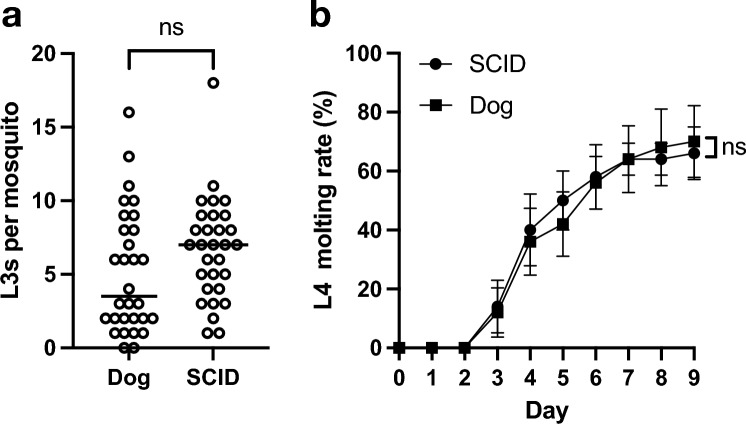
Developmental ability of microfilariae maintained in SCID mice. (**a**) Number of the L3 in individual mosquito fed with microfilaria-infected blood obtained from dog or SCID mice (n = 30). ^ns^*p* > 0.05 (Mann–Whitney test). (**b**) In vitro L3 to L4 molting assay. L3 obtained from mosquitoes fed with microfilaria-infected blood obtained from SCID mice or infected dog was used. ^ns^*p* > 0.05 (Mann–Whitney test on day 9). *SCID* severe combined immunodeficiency.

To test microfilariae infectivity to mosquitoes, mosquitoes were fed microfilariae obtained from either SCID mice or a dog. There was no significant difference in the number of L3s between SCID mouse and dog groups (*p* = 0.0742), indicating the infectivity of the microfilaria (Fig. 3a). To evaluate the developmental ability of L3 to L4, L3s isolated from mosquitoes fed with microfilariae obtained from SCID mice or dogs were used in the L4 developmental assay. On day nine, there was no significant difference in the L4 developmental ratio between the SCID mouse and dog groups (*p* = 0.6825, Fig. 3b).

### Microfilariae maintained in SCID mice displayed normal viability and responded normally to ivermectin in vitro

**Figure 4:**
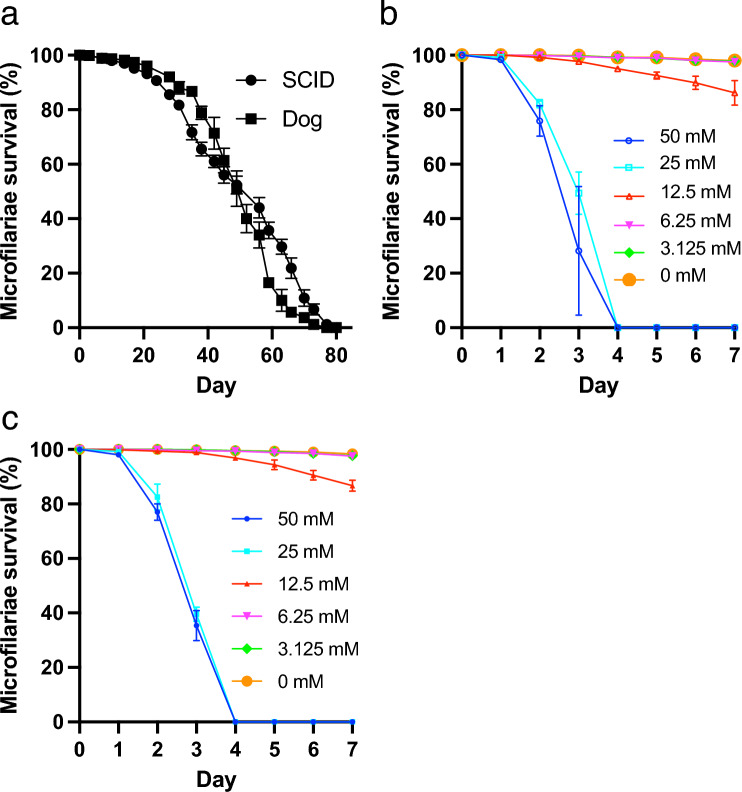
Viability and response to ivermectin in vitro by microfilaria maintained in SCID mice. (**a**) Survival rate of the microfilaria in vitro. Microfilariae obtained from SCID mice or an infected dog were monitored in vitro. Microfilaria survival was statistically compared from day 14 to day 70 every 2 weeks. There was no significant difference (*p* > 0.05, Mann–Whitney test). Anthelmintic ivermectin response assay of the *D. immitis* microfilaria obtained from the SCID mice (**b**) or infected dog (**c**). The survival rate of microfilaria was monitored every day. Microfilariae survival between the SCID and Dog groups was statistically compared on day 3. There was no significant difference at all concentration (*p* > 0.05, unpaired t-test). *SCID* severe combined immunodeficiency.

Microfilariae collected from SCID mice or the source dog were cultured in vitro to validate their viability. There was no significant difference in microfilariae survival between SCID mice and the source dog (*p* = 0.1000, Fig. 4a). Both groups survived for ten weeks (Fig. 4a). Microfilariae from SCID mice (Fig. 4b) and the source dog (Fig. 4c) were cultured in vitro with ivermectin to assess their response to antiparasitic medication. Those from SCID mice exhibited a survival pattern similar to that observed after ivermectin treatment in dogs. There was no significant difference in microfilaria survival between the SCID mouse and source dog groups on day 3 (Fig. 4b,c).

### The SCID mouse model proved invaluable when isolating clinical samples and studying mosquito infection

**Figure 5:**
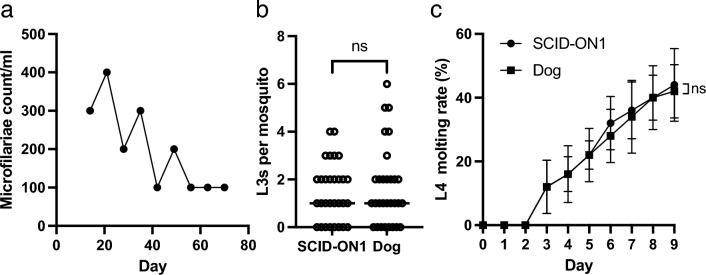
The SCID mouse as a rodent model for the isolation of *D. immitis* microfilaria from clinical sample. Microfilariae (ON1) were isolated from clinical samples and inoculated into a SCID mouse 3 days after blood collection. (**a**) Microfilaremia in SCID mice inoculated with *D. immitis* ON1 microfilaria. (**b**) L3s in individual mosquitoes fed with microfilariae blood (1.2 microfilariae/mL) of a SCID mouse inoculated with ON1 *D. immitis* microfilariae or an infected dog. Bars indicate median. ^ns^*p* > 0.05 (Mann–Whitney U test). (**c**) In vitro L3–L4 molting assay. L3 obtained from mosquitoes fed microfilaria-infected blood obtained from a SCID-ON1mouse, or an infected dog was used. ^ns^*p* > 0.05 (Mann–Whitney U test on day 9). *SCID* severe combined immunodeficiency.

A SCID mouse was inoculated with microfilariae from a clinical sample (ON1). The SCID-ON1 mouse exhibited microfilaremia for 10 weeks (Fig. 5a). Subsequently, the SCID-ON1 mouse was sacrificed by cardiac puncture. Mosquitoes were then fed the blood of the SCID-ON1 mouse to determine the ability of L3 larvae to develop, and they were compared to mosquitoes fed microfilariae from an infected dog. The number of L3 larvae was not significantly different between the SCID-ON1 and dog groups (*p* = 0.9554, Fig. 5b). Furthermore, there was no significant difference in the developmental ability of L3 to L4 between the SCID-ON1 and dog groups at nine dpi (*p* = 0.9921, Fig. 5c).

### The SCID mice model was valuable for assessing the recovery and fitness of cryopreserved microfilariae

**Figure 6:**
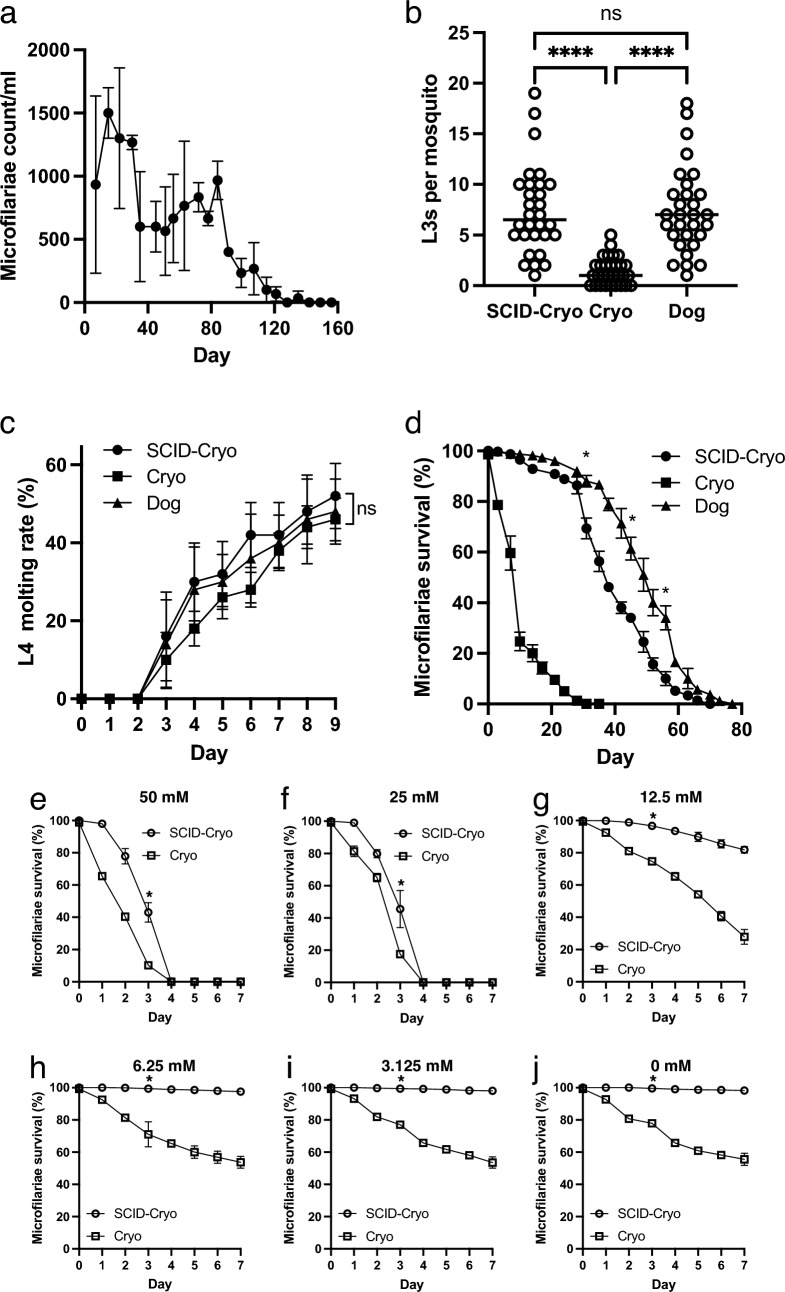
Fitness of the cryopreserved microfilaria from the SCID mouse. (**a**) Microfilariaemia of the SCID mice inoculated with cryopreserved microfilariae (n = 3). (**b**) L3 number of the individual mosquitoes fed with microfilariae collected from the SCID mice blood inoculated with cryopreserved microfilariae (SCID-Cryo), cryopreserved microfilariae (Cryo), or collected from an infected dog (Dog). ****p < 0.0001, ^ns^*p* > 0.05 (Kruskal–Wallis test). (**c**) In vitro L3-to-L4 molting assay. L3 obtained from the mosquitoes fed with blood of the SCID mice inoculated with cryopreserved microfilariae (SCID-Cryo), fed with cryopreserved microfilaria (Cryo), or from the mosquitoes fed with blood collected from an infected dog (Dog). ^ns^*p* > 0.05 (one-way ANOVA on day 9). (**d**) Microfilariae viability assay in vitro. Survival rate of microfilariae obtained from SCID mice inoculated with cryopreserved microfilariae (SCID-Cryo), those from an infected dog (Dog), or cryopreserved microfilariae (Cryo). Microfilaria survival was statistically analyzed every 2 weeks by one-way ANOVA. *p < 0.05 both between SCID-Cryo and Cryo, and SCID-Cryo and Dog. (**e–j**) Anthelmintic ivermectin responsible assay of the *D. immitis* microfilaria obtained from the SCID mice inoculated with cryopreserved microfilariae or cryopreserved microfilariae. (**e**) 50 mM, (**f**) 25 mM, (**g**) 12.5 m, (**h**) 6.25 mM, (**i**) 3.125 mM and (**j**) 0 mM. Microfilariae survival was monitored every day. Microfilaria survival on each ivermectin concentration between the SCID and Dog groups was statistically compared on day 3. There were significant differences in all concentrations (*p < 0.05, unpaired t-test). *SCID* severe combined immunodeficiency.

SCID mice were inoculated with cryopreserved or fresh microfilariae collected from an infected dog. Those inoculated with the cryopreserved microfilariae exhibited microfilaremia for 15 weeks (Fig. 6a). To determine the infectivity of cryopreserved microfilariae recovered from SCID mice, mosquitoes were fed microfilariae isolated from SCID mice inoculated with cryopreserved microfilariae (SCID-Cryo), cryopreserved microfilariae (Cryo), and non-cryopreserved microfilariae obtained from a *D. immitis*-infected dog (Dog). The number of L3 larvae did not differ significantly between the SCID-Cryo and Dog groups (*p* > 0.9999, Fig. 6b). The SCID-Cryo (*p* < 0.0001) and Dog (*p* < 0.0001) groups had significantly more L3 larvae than the Cryo group (*p* = 0.9921, Fig. 6b). On day 9, there was no significant difference in the developmental ability of the L3 larvae obtained from mosquitoes fed SCID-Cryo, Cryo, or Dog groups (*p* = 0.8339, Fig. 6c). The viability of the SCID-Cryo microfilariae was determined using in vitro cultivation. SCID-Cryo microfilariae survived significantly higher than Cryo microfilariae on day 14 (*p* < 0.0001), 28 (*p* < 0.0001), 42 (*p* < 0.0001) and 56 (*p* = 0.0200) (Fig. 6d). The reactions of SCID-Cryo and Cryo microfilariae to ivermectin were investigated (Fig. 6e–j). SCID-Cryo microfilariae (Fig. 6j) had a survival pattern comparable to that of fresh microfilariae collected from a dog (Fig. 4c) or SCID mice inoculated with non-cryopreserved microfilaria (Fig. 4b). At all ivermectin concentrations, including the control group, the SCID-Cryo microfilariae group outlived the Cryo group on day 3 (Fig. 6e–j).

## Discussion

This study demonstrated the usefulness of an in vivo rodent *D. immitis* microfilaremia model. SCID mice could maintain microfilaremia in peripheral blood for up to 7 months and at least 10 months in cardiac blood. These results suggest that SCID mice are effective for the long-term maintenance of *D. immitis* microfilaremia.

The lack of rodent models complicates canine heartworm studies. Establishing an in vivo model of *D. immitis* requires a well-equipped facility capable of rearing dogs or ferrets to isolate microfilariae. In 1983, Grieve et al.^12^ developed a mouse model of *D. immitis* microfilaremia. They used immunodeficient BALB/cA mice and sublethal gamma-ray irradiation to induce microfilaremia in the peripheral blood that lasts for 5 weeks. This finding motivated us to investigate the potential of SCID mice for the long-term maintenance of *D. immitis* microfilariae. Furthermore, the utility of SCID mice as a model for L3/L4 infection with canine filariasis has been reported^17,18^. SCID mice have been validated as a full-development model of *Brugia malyai* and microfilaria production^19^. It has been reported that microfilariae of *Onchocerca lienalis* are maintained for more than 100 days in SCID mice^20^. These reports led us to hypothesize that SCID mice may serve as a model for dog *D. immitis* microfilaraemia. Moreover, we investigated whether splenectomy could prolong microfilaremia, hypothesizing that the spleen, as a component of the immune system responsible for eliminating cellular waste from the blood, would play a role in this process^21^. Although we were unable to establish the efficacy of splenectomy in prolonging the duration of microfilaremia, SCID mice proved valuable for the long-term maintenance of microfilaremia, which is consistent with our hypothesis. The majority of microfilariae in dogs infected with *D. immitis* live in their pulmonary capillaries^22,23^. Microfilariae were detected in SCID mice for a longer period of time in cardiac puncture blood than in peripheral blood. This shows that the dynamics of microfilariae in SCID mice are similar to those observed in dogs. Microfilaremia levels in SCID mice exhibited periodicity, with an increase at night. This trend was comparable to that seen in dogs^24^. These findings suggested that SCID mice can replicate the dynamics of microfilariae in their bodies, making them valuable models for studying the periodicity of *D. immitis* microfilariae. However, additional validation is required.

The normalcy of microfilariae is crucial when using microfilariae maintained in SCID mice to discover new drugs or study mosquito transmission mechanisms. We conducted in vitro cultivation, mosquito infectivity, L4 development, and ivermectin reactivity assays. The in vitro cultivation of microfilariae is crucial for drug screening^25^. In our study, microfilariae isolated from SCID mice had a survival rate comparable to that of microfilariae isolated from infected dogs. This finding suggests that the lifespan of microfilariae was not shortened in SCID mice, implying that the microfilarial environment in SCID mice is comparable to that in dogs. In the ivermectin reactivity assay, microfilariae isolated from SCID mice reacted similarly to microfilariae recovered from dogs. This suggests that maintaining microfilariae in SCID mice does not affect their metabolism or homeostasis in response to ivermectin. As a result, SCID mice are regarded as a valuable source of microfilariae for drug screening or macrocyclic lactone resistance assays, although only one compound was tested in this study. This finding further supports the idea that the microfilarial conditions in SCID mice are nearly identical to those in dogs.

To determine the infectivity and developmental potential of microfilariae, we infected *Ae. aegypti* mosquitoes with microfilariae from SCID mice and counted the number of L3 larvae in each mosquito. Our results show that microfilariae isolated from SCID mice have normal infectivity when compared to those recovered from infected dogs. Additionally, L3 larvae derived from SCID mice displayed normal developmental ability to advance to the L4 stage in vitro. These results suggest that SCID mice can serve as an important source of microfilariae for mosquito infection experiments and L3 to L4 developmental tests for the development of innovative anti-heartworm preventive medicines.

The use of microfilariae derived from clinical samples is crucial for research purposes such as macrocyclic lactone resistance^26^. Maintaining the viability of microfilariae between clinical sample collection and the experimental setting is critical; however, long-term preservation presents challenges. Microfilariae can be cultured in vitro for up to 45 days^10^. Alternatively, cryopreservation may be used. However, the viability of cryopreserved microfilariae is frequently low. To address this, we used SCID mice to isolate and maintain microfilariae from veterinary clinical samples. We successfully established that SCID mice can be used to isolate and maintain microfilariae obtained from clinical samples, even when the blood sample is refrigerated for three days before being inoculated into the SCID mouse. Notably, ON1 microfilariae showed normal infectivity in mosquitoes and the developmental ability to proceed to the L4 stage. These findings highlight the efficacy of the SCID mouse model for isolating clinical microfilariae samples and suggest that it has the potential to improve the use of clinical sample-derived microfilariae in heartworm research.

However, the viability of cryopreserved microfilariae after thawing remains a substantial challenge^11^. In our earlier investigation, we found that thawed microfilariae had inferior fitness, despite approximately 80% of the microfilariae surviving 3 days after thawing in vitro^9^. Furthermore, the number of L3 larvae recovered from mosquitoes infected with cryopreserved microfilariae was approximately one-third of that recovered from mosquitoes infected with an equal number of non-cryopreserved fresh microfilariae^9^. In this study, we used SCID mice to recover the cryopreserved microfilariae. Notably, the cryopreserved microfilariae recovered from SCID mice had comparable infectivity and L4 developmental ability to fresh microfilariae collected from experimentally infected dogs. Furthermore, these cryopreserved microfilariae showed a similar survival curve in macrocyclic lactone resistance when compared to fresh microfilariae collected from experimentally infected dogs. These findings indicate that the microfilariae selected from cryopreserved samples retain their fitness and can be effectively recovered using SCID mice. This hypothesis is reinforced by the fact that cryopreserved microfilariae recovered from SCID mice survived longer in vitro than directly thawed cryopreserved microfilariae. Therefore, the combination of cryopreservation and recovery in SCID mice appears promising, particularly for research on macrocyclic resistance using microfilariae isolated from clinical samples. This approach effectively addresses the challenges of long-term preservation and microfilariae fitness in research involving clinical samples.

Recent studies have established mouse models for L4 development in *D. immitis* using NOD-, NSG-, or NXG immunodeficient mice^17,18^. This study proposes a microfilaremia model for *D. immitis* using SCID mice. Developing a comprehensive rodent model of *D. immitis* infection shows potential for furthering heartworm research, particularly in terms of creating novel preventative strategies that overcome macrocyclic lactone resistance. Furthermore, these mouse models have the potential to improve animal welfare by minimizing the use of experimental companion animals, such as dogs and cats.

In this study, we established a dog heartworm microfilaremia model and demonstrated that our mouse model is useful for the source of microfilariae for the research, such as drug screening or transmission mechanism. For the drug research, in vivo infection model is useful. Our SCID mouse model may be useful for the in vivo study, although we could not determine in vivo drug administration test in this study. We hope that our SCID mouse model could contribute to the development of the novel anthelmintics in a future study.

## Data Availability

The datasets generated during and/or analyzed during the current study are available from the corresponding author on reasonable request.
